# How noise correlations impact the amount of information in superior colliculus: the analysis of a population with shared receptive fields

**DOI:** 10.1186/1471-2202-15-S1-P72

**Published:** 2014-07-21

**Authors:** Saba Farbodkia, Kelly Shen, Gregory Day, Martin Paré

**Affiliations:** 1 Center for Neuroscience Studies, Queen’s University, Kingston, Ontario, K7L 3N6, Canada

## 

Superior colliculus (SC) visuomovement neurons recorded while monkeys perform two versions of visual search task with different difficulty levels have discriminating ability that exceeds the monkey’s discriminating ability [1]. This suggests that there is substantial noise added to this neuronal population. We investigated the effect of noise correlation as a possible limiting factor on the population’s overall discrimination ability. We simultaneously recorded from pairs of neurons during the simpler version of the task, and quantified the noise correlations for each pair. As expected noise correlations were mostly positive for pairs that shared the same receptive field (average= 0.14), and they mostly had the same sign as the signal correlations. Theoretically, this should reduce the amount of information that the pairs carry about the stimulus. The dependence of noise correlations on the stimuli (0.14 for target, 0.13 for distracters) was not statistically significant (p= 0.7); therefore, we didn’t expect that information be significantly added to most of pairs. We investigated the overall impact of noise correlations on the amount of information that each pair carried, by and quantifying the amount of information that the pair carried, when the noise correlations were ignored. The impacts were negligible for each pair. However, it is possible that at the level of population, the impact be different from what we observed for the pairs, due to accumulation of small amounts of correlation. To completely rule out the possibility of limiting effects of noise correlations on growth of information in the population, we investigated if the impacts would be similar in a larger population. We simulated a population of neurons with their responses sharing the basic discharge properties of our recorded neurons during each version of the task, and incorporated randomly assigned noise correlations drawn from a normal distribution with its mean and standard deviation taken from what we had obtained from the simultaneously recorded neurons. Our results so far show that at least for the simpler version of the task, where the signal-to-noise ratio is generally higher, the noise correlations do not have a significant effect, even when the population size grows large enough to saturate the mutual information between the signal and neural responses to its maximum possible amount. Therefore, it can be suggested that noise correlations do not play an important role in limiting the amount of information in a population of SC neurons during a feature visual search task.
